# Recent advances in lincosamide biosynthetic studies

**DOI:** 10.1038/s41429-025-00884-x

**Published:** 2025-11-25

**Authors:** Yi Yang, Takahiro Mori

**Affiliations:** 1 Graduate School of Pharmaceutical Sciences, The University of Tokyo, Tokyo, Japan; 2 Collaborative Research Institute for Innovative Microbiology, The University of Tokyo, Tokyo, Japan

**Keywords:** Enzymes, Biosynthesis

## Abstract

Lincomycin A and celesticetin are representative members of the lincosamide class of clinically used antibiotics produced by *Streptomyces* species. Their distinctive chemical architectures arise from atypical biosynthetic gene clusters that lack well observed signature genes, and since the complete determination of the lincomycin A biosynthetic pathway, current research has focused on the genetic manipulation of regulatory elements and the protein engineering of biosynthetic enzymes. This review summarizes recent advances in elucidating the transcriptional regulation of lincosamide biosynthetic gene clusters and the structure–function relationships and engineering of their biosynthetic enzymes.

## Introduction

Historically, natural products have been an important source of lead molecules for drug discovery. This is especially so for antibiotics, whereby the discovery and subsequent development of penicillin in the early 1940s heralded the golden age of antibiotics, spanning from the early 1940s to the late 1960s. However, since the 1970s, the dramatic reduction in the discovery rate of novel compound classes and the emergence of multi-drug resistance in pathogens have challenged scientists and clinicians to devise new strategies in the fight against infectious diseases [1, 2]. Accordingly, significant efforts have been made toward the chemical and enzymatic modification of naturally derived antibiotics, leading to a spectrum of semi-synthetic compounds with improved antimicrobial activities over their parent molecules. Additionally, facilitated by new technologies in the post-genomic era, explorations of novel biosynthetic gene clusters with unusual features and organizations promise to uncover new lead scaffolds that were previously overlooked and provide biocatalysts for the generation of unique analogs [3–5].

**Fig. 1:**
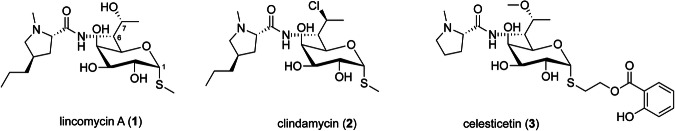
Chemical structures of natural and semi-synthetic lincosamides

An important class of antibiotics that has emerged from these approaches is the lincosamides. Lincomycin A (**1**) and its semisynthetic derivative, clindamycin (**2**), have been used for over five decades to treat infections caused by Gram-positive bacteria and mycoplasmas, particularly in patients with penicillin hypersensitivity [6, 7]. These compounds inhibit bacterial protein synthesis by binding to the peptidyltransferase domain of the 50S ribosomal subunit. Structurally, lincosamides consist of a thiooctose core conjugated at the C6 position to either proline or an alkylproline moiety, with an additional *S*-alkyl substitution at the C1 position [8]. Lincomycin A, produced by *Streptomyces lincolnensis*, incorporates *N*-methylated trans-4-propyl-L-proline (PPL) at C6 and carries a C1 *S*-methyl group. In contrast, celesticetin (**3**), isolated from *Streptomyces caelestis*, contains *N*-methylproline at C6, a C7 methoxy group, and a salicylic acid moiety tethered to the C1 sulfur atom via a two-carbon linker (Fig. 1) [9, 10].

**Fig. 2:**
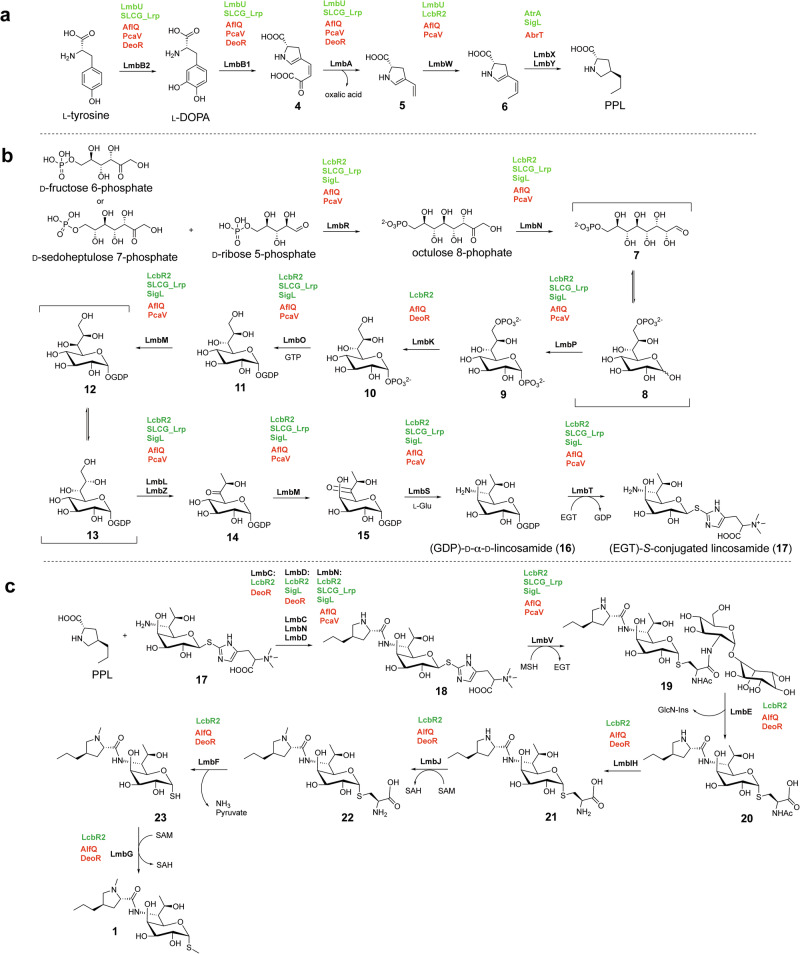
Regulation of the lincomycin A biosynthetic pathway. **a** PPL is biosynthesized via **4** – **7** from L-DOPA which is in turn derived from L-tyrosine. **b** Octulose 8-phophate formation and subsequent derivatization into thiooctose via **7** – **16**. **c** condensation of **17** with PPL and *S*-functionalization of **18** to generate lincomycin A via **19** – **23**. Positive regulators are highlighted in green and negative regulators are highlighted in red

The biosynthetic pathway of lincomycin A was confirmed in 2020, by the identification of the functions of LmbM, LmbL, CcbZ (an LmbZ homologue), and CcbS (an LmbS homologue) in catalyzing the conversion of GDP-octose (**15**) to GDP-D-α-D-lincosamide (**16**) (Fig. 2) [11]. Likewise, analyses of celesticetin biosynthesis have elucidated most of its pathway. Collectively, the biosynthesis and pharmacodynamics of lincomycin A, celesticetin, and their semisynthetic analogs are now well established and have been comprehensively reviewed in the literature [9, 11, 12]. Research on the regulatory mechanisms controlling the expression of lincosamide biosynthetic gene clusters (BGCs) has been ongoing for over a decade. More recently, advances in molecular biology and systems approaches have deepened our understanding of these networks and facilitated their manipulation for strain improvement. In parallel, structure–function analyses of key biosynthetic enzymes have enabled protein engineering strategies to generate novel lincosamide analogs. This review highlights recent progress in the exploitation of lincosamide biosynthetic genes for enhanced antibiotic production.

## The *lmb* and *ccb* BGCs

**Fig. 3:**
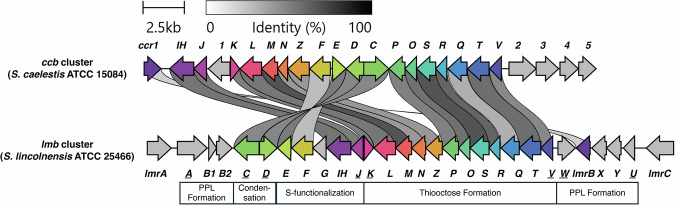
The *lmb* and *ccb* BGCs. Homologous genes between the *lmb* and *ccb* clusters are color coded, and the gene leading each lmb operon is underlined

The 35-kb lincomycin biosynthetic gene cluster (BGC) was first cloned and characterized from the industrial strain *Streptomyces lincolnensis* 78-11 [13]. It comprises 26 biosynthetic and regulatory genes (*lmb*) and 3 resistance genes (*lmr*) (Fig. 3 and Table 1). Aside from *lmrA* and *lmrC*, which flank the cluster, the remaining 27 open reading frames are organized into 8 operons headed by *lmbA*, *lmbC*, *lmbD*, *lmbJ*, *lmbK*, *lmbV*, *lmbW* and *lmbU*, respectively. The genes in these operons are loosely grouped based on their biosynthetic functions: alkylproline formation, thiooctose formation, condensation coupling, and *S*-alkyl functionalization (Table 2) [14]. In the celesticetin (*ccb*) BGC from *Streptomyces caelestis*, the homologs of the *lmb* genes responsible for thiooctose generation and condensation are highly conserved. In contrast, this cluster contains only one resistance gene, *ccr1*, which is homologous to *lmrB* (Fig. 3 and Table 1) [14]. The *lmb* and *ccb* clusters are atypical BGCs in that they lack signature enzymes such as non-ribosomal peptide synthases (NRPSs) and polyketide synthases (PKSs). Instead, their core scaffold is assembled by unusual condensation enzymes that catalyze the amide bond formation between an amino acid and a thiooctose moiety (Fig. 2). Another distinctive feature of the lincomycin BGC is its reliance on three different self-resistance mechanisms. The *lmrA*, *lmrB*, and *lmrC* genes encode an MFS transporter, a 23S rRNA methyltransferase, and an ATP-binding cassette-F (ABCF) ATPase, respectively [13, 15]. Each of these mechanisms can individually provide a different degree of protection against lincosamide antibiotics.

**Table 1:** Gene products of the *lmb* and *ccb* gene clusters and their respective functions

Lmb	Ccb	Function	Lmb	Ccb	Function
LmrA	-	MFS transporter	O	O	Nucleotidyltransferase
A	-	Hydrolase	S	S	Transamidase
B1	-	L-DOPA 2,3-dioxygenase	R	R	Transaldolase
B2	-	L-tyrosine hydroxylase	Q	Q	*N*-deacetylase
C	C	Adenylation domain	T	T	*S*-glycosyltransferase
D	D	Transpeptidase	V	V	*S*-glycosyltransferase
E	E	Amidase	W	-	*C*-methyltransferase
F	F	PLP-dependent enzyme	LmrB	Ccr1	23S rRNA methyltransferase
G	-	*S*-methyltransferase	X	-	Epimerase
IH	IH	*N*-deacetylase	Y	-	Reductase
J	J	*N*-methyltransferase	U	-	Transcription factor
K	K	Phosphatase	LmrC	-	ABCF ATPase
L	L	Dehydrogenase	-	1	Acyltransferase
M	M	Epimerase	-	2	Acyl-CoA ligase
N	N	Isomerase (C-terminal domain); Ppant domain (N-terminal domain)	-	3	Salicylate synthase
Z	Z	NAD(P)-dependent oxidoreductase	-	4	*O*-methyltransferase
P	P	Kinase	-	5	Oxidoreductase

**Table 2:** Organization and regulation of lincosamide biosynthetic operons

Operon	Biosynthetic Phase	Direct Regulators			
		Positive	Class	Negative	Class
*lmbAB1B2*	PPL formation (early stage)	LmbU SLCG_Lrp	LmbU Lrp	AflQ PcaV DeoR	TCS MarR DeoR
*lmbC*	Condensation	LcbR2	MarR	DeoR	DeoR
*lmbD*	Condensation	LcbR2 SigL	MarR σ factor	DeoR	DeoR
*lmbJIHGFE*	S-functionalization	LcbR2	MarR	AflQ DeoR	TCS DeoR
*lmbK*	Thiooctose formation	LcbR2	MarR	AflQ DeoR	TCS DeoR
*lmbVTQRSOPZNML*	Thiooctose formation	LcbR2 SLCG_Lrp SigL	MarR Lrp σ factor	AflQ PcaV	TCS MarR
*lmbW*	PPL formation (late stage)	LmbU LcbR2	LmbU MarR	AflQ PcaV	TCS MarR
*lmbUXY-lmrB*	PPL formation (late stage)	AtrA SigL	TetR σ factor	AbrT	TetR

## Regulation of lincosamide BGC expression

Lincosamides are chemically complex and are therefore produced industrially through bacterial fermentation [16]. These natural products are further modified into semi-synthetic analogs, such as clindamycin. Following the elucidation of the lincomycin biosynthetic pathway, research has increasingly shifted toward understanding and manipulating the transcriptional regulation of the cluster, with the practical goal of developing overproducing strains for improved antibiotic yields. Such regulation is typically mediated by transcription factors that bind to cis-regulatory elements (CREs) near target genes, leading to transcriptional activation or repression of the locus and its associated operon (Fig. 2 and Table 3) [16, 17]. These interactions can be investigated by in vitro techniques such as electrophoretic mobility shift assays (EMSAs), which visualize binding between proteins and gene elements [16, 17]. In general, the overexpression of positive regulators such as LmbU, SLCG_Lrp, and SigL, as well as the deletion of negative regulators such as AflQ, PcaV, DeoR, and AbrT, increased the lincomycin titers from *S. lincolnensis* [17–23]. However, some mutant strains showed altered growth and morphology, emphasizing the importance of clarifying the regulatory network of lincomycin biosynthesis to facilitate future genetic engineering [18, 19, 21, 22]. Additionally, transcription factors can exert indirect control over biosynthetic genes by modulating the expression of other regulators that act directly on the genes of interest [15, 24–29]. This layered regulation forms multi-branched signaling cascades that frequently involve other classes of transcription regulators. Because the interplay among transcription factors during the indirect regulation of secondary metabolism is highly complex and difficult to quantify, this section focuses on newly discovered mechanisms underlying the direct transcriptional control of *lmb* cluster genes in the context of the different stages of lincomycin biosynthesis.

**Table 3:** CREs of transcription regulators in *lmb* operons

Type	Regulator	Class	Target Operons	Binding Motifs (5’→3’)	Reference
Positive	LmbU	LmbU	*lmbAB1B2* *lmbW*	CGCCGGCG	[17]
	SLCG_Lrp	Lrp	*lmbAB1B2* *lmbVTQRSOPZNML*	GGAGAATTTCCCTCATGGAT	[18]
	AtrA	TetR	*lmbUXY-lmrB*	GGAANNNNNNNTTCC	[32]
	LcbR2	MarR	*lmbC* *lmbD* *lmbJIHGFE* *lmbK* *lmbVTQRSOPZNML* *lmbW*	TTGCCnnnnnCAA	[33]
	SigL	σ factor	*lmbD* *lmbVTQRSOPZNML*	N.D.	-
Negative	AlfQ	TCS	*lmbAB1B2* *lmbJIHGFE* *lmbK* *lmbVTQRSOPZNML* *lmbW*	GTNAC-N_6_-GTNAC	[20]
	PcaV	MarR	*lmbAB1B2* *lmbVTQRSOPZNML* *lmbW*	TCAGnnnnCnnA	[21]
	DeoR	DeoR	*lmbAB1B2* *lmbC* *lmbD* *lmbJIHGFE* *lmbK*	CGATCR	[22]
	AbrT	TetR	*lmbUXY-lmrB*	CGCGTACTCGTA CGTACGATAGCT	[23]

## Cluster-situated regulators: LmbU and LmrC

The first regulatory element identified in the lincomycin BGC was LmbU in 2018. LmbU is a member of a novel class of regulatory proteins widely distributed in actinomycetes [17]. In vitro EMSA experiments showed that LmbU binds directly to CREs located upstream of *lmbA* and *lmbW*. This binding activates the expression of the *lmbAB1B2* and *lmbW* operons, which are involved in the synthesis of PPL, the alkylproline subunit of lincomycin A. More recently, LmbU targets outside the *lmb* cluster have been identified through genomic screening for the palindromic LmbU-binding motif, and their regulation has been confirmed by comparative transcriptomic analyses and EMSAs [30]. Among these, the products of three genes, *SLINC_0469*, *SLINC_1037*, and *SLINC_8097*, act as negative regulators of lincomycin biosynthesis, providing a basis for future studies on the full regulatory cascade mediated by LmbU [30].

LmrC belongs to the ABCF family of proteins that binds to and controls the functional state of the 50S ribosomal subunit, where its antibiotic resistance domain obstructs antibiotic binding to ribosomes [13, 15]. However, compared to the MFS transporter LmrA, which performs antibiotic efflux, and the 23S rRNA methyltransferase LmrB, which reduces ribosome susceptibility to lincomycin, LmrC does not contribute significantly to the overall lincosamide resistance phenotype [15]. Notably, LmrC has been characterized as a regulatory ABCF protein in lincomycin biosynthesis, functioning as an antibiotic sensor that increases lincomycin production in response to lincosamide exposure. Specifically, a premature terminator in the 5’ untranslated region of *lmrC* inhibits LmrC expression in the absence of ribosome-targeting antibiotics. Lincosamide binding to ribosomes converts the terminator into an antiterminator conformation, thus alleviating the attenuation of LmrC expression. Subsequently, LmrC was shown to upregulate *lmbU* in an ATPase activity-dependent manner, as overproduction of ATPase-deficient variants of LmrC failed to induce *lmbU* expression [15]. Additionally, binding of LmrC to the CRE of *lmbU* activates transcription of the entire *lmbUXY-lmrB* operon, which encodes the genes *lmbX* and *lmbY* involved in PPL production. As LmbU in turn upregulates LmbA, LmbB1, LmbB2 and LmbW, the LmrC-LmbU signaling cascade likely represents a regulatory axis that specifically enhances the supply of the alkylproline building block during lincomycin biosynthesis.

## External regulators

In 2019, SLCG_2919, a tetracycline repressor (TetR)-type transcription factor located outside of the lincomycin BGC, was reported to repress lincomycin production in *S. lincolnensis* by directly inhibiting cluster gene transcription [31]. Since then, transcription factors from the multiple antibiotic resistance regulator (MarR), leucine-responsive regulatory protein (Lrp), and deoxyribose operon repressor (DeoR) families, as well as other regulators such as sigma factors and two-component signal transduction systems (TCSs), have been implicated in the direct transcriptional control of one or more operons of the lincomycin BGC, as summarized in Table 2. Positive external regulators of lincomycin biosynthesis include members of the TetR, MarR, Lrp, and sigma factor families. Among them, AtrA, a TetR-type transcription factor, directly activates the *lmbUXY-lmrB* operon, upregulating the PPL biosynthesis in a manner similar to LmrC [32]. The Lrp member SLCG_Lrp exhibits a broad regulon, binding not only to the promoter of *lmbU* but also to those of *lmbA* and *lmbV*, thus contributing to the biosynthesis of both PPL and methyllincosamide (MTL), the thiooctose moiety of lincomycin A [18]. Likewise, the sigma factor SigL regulates three operons by binding to the promoters of *lmbU*, *lmbV*, and *lmbD* [19]. Through these interactions, SigL extends its regulatory role beyond the synthesis of individual building blocks (such as PPL) to the condensation step between the PPL and MTL subunits. Meanwhile, the MarR-family regulator LcbR2 binds to nearly all operon CREs except those for *lmbA* and *lmbU*, exerting control over every biosynthetic phase and representing the first direct activator of *S*-alkyl functionalization genes, encoded by the *lmbJIHGFE* operon [33].

Negative direct regulators of lincomycin biosynthesis have also been identified. These include PcaV, AbrT, and DeoR, belonging to the MarR, TetR, and DeoR transcription factor families, respectively, as well as the two-component system AflQ, composed of the histidine kinase AflQ2 and the response regulator AflQ1 [20–23]. In contrast to the relatively restricted regulons of positive regulators, negative regulators typically exert broader effects on the entire lincomycin biosynthetic pathway by repressing at least one operon involved in each stage [20–23]. This is consistent with the canonical functions of the MarR and DeoR transcription factors, as well as those of the TCS proteins, which act as global repressors. An exception is the TetR-type regulator AbrT, which binds specifically to *lmbU* and may regulate late-stage PPL biosynthesis in cooperation with the positive regulators LmrC and AtrA [23]. However, the interplay among these regulators has yet to be thoroughly elucidated, necessitating further investigation to clarify potential feedback mechanisms, in which positive and negative regulators influence each other to maintain antibiotic biosynthesis at a consistent level.

## Structural analysis and enzyme engineering of lincosamide biosynthetic enzymes

In addition to studies on regulatory factors aimed at improving antibiotic yields, the molecular bases of lincomycin and celesticetin biosynthesis have also garnered significant scientific interest. Here, the high homology observed between key biosynthetic enzymes from the *lmb* and *ccb* BGCs accentuates the potential for the combinatorial biosynthesis of chimeric products [14]. Moreover, analyses of substrate specificities and engineering of product specificities hold promise for developing biocatalysts capable of generating non-natural lincosamide analogs. An early milestone in this direction was the structural and mechanistic investigation of the *N*-methyltransferase CcbJ in 2014, where the structural analysis of its active site suggested the potential for substrate promiscuity [34]. Subsequently, structure–function studies have been extended to other enzymes such as CcbD, LmbT, and the homologous pair CcbF/LmbF, thereby broadening our understanding of the catalytic diversity underlying lincosamide biosynthesis.

## Structure-function and substrate scope analyses of LmbT and CcbD

**Fig. 4:**
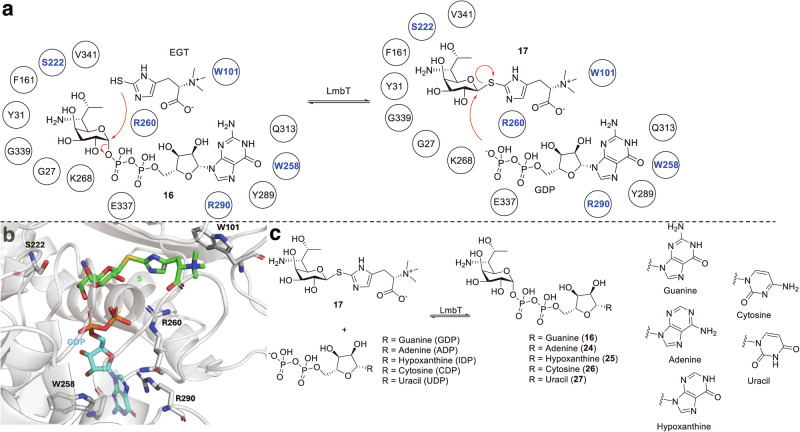
Structure-function analysis and substrate specificity of LmbT. **a** Proposed catalytic mechanism of LmbT. Key residues for substrate specificity are highlighted in blue. **b** Active site of LmbT in the complex structure with **5** and GDP (PDB ID: 8ILA). **c** Substrate promiscuity of LmbT

One of the distinctive structural features of lincosamides is the presence of a sulfur atom-containing thiooctose moiety. The incorporation of sulfur occurs via the generation of an ergothioneine (EGT)-*S*-conjugated lincosamide (**17**) from GDP-D-α-D-lincosamide (**16**) and EGT. This step is catalyzed by the *S*-glycosyltransferases LmbT and CcbT in the lincomycin A and celesticetin biosynthetic pathways, respectively. In 2023, independent in vitro studies of LmbT collectively revealed an S_N_2-like mechanism, in which the interconversion between the EGT- and GDP-conjugated forms occurred, and the nucleophilic attack by either the thiol group of EGT or the phosphate group of GDP from the opposite faces led to the inversion of the C1 stereochemistry of the thiooctose moiety (Fig. 4a) [35, 36]. Crystal structures of LmbT confirmed its similarity to type-B glycosyltransferases, consisting of two Rossman domains separated by a cleft that forms the active site. Substrate binding to the active site induces large conformational changes to accommodate the ligands [36].

Structural and mutagenesis studies of LmbT identified Arg260 as a key residue that interacts with both the diphosphate group of GDP and the carboxylic acid moiety of EGT. EGT recognition is further stabilized by a cation–π interaction between its trimethylamine group and Trp101. The guanosine moiety of GDP is bound in a hydrophilic pocket composed of Trp258, Thr289, Arg290, and Gln313. Within this pocket, the cation-π and T-shaped π-π interactions involving Arg290 and Trp258, respectively, with the purine ring suggest promiscuity towards other nitrogenous bases (Fig. 4b). Consistently, in vitro experiments showed that LmbT accepts adenosine diphosphate (ADP), inosine diphosphate (IDP), cytidine diphosphate (CDP), and uridine diphosphate (UDP) as alternative substrates to generate the corresponding analogs of GDP-D-α-D-lincosamide (**24**-**27**) (Fig. 4c) [36]. Purine substrates were favored over pyrimidine substrates, whereas thymidine diphosphate (TDP) was not accepted due to its hydrophobic C5 methyl group. Although the active site is large enough to accommodate other GDP-sugars, the interactions within a sub-pocket composed of Gly27, Tyr31, Phe161, Ser222, Val341, and Gly339 with the C8 side chain of the sugar moiety, together with the specific interaction between Ser222 and the C7 hydroxyl group, enforce the strict specificity of LmbT for thiooctose substrates.

**Fig. 5:**
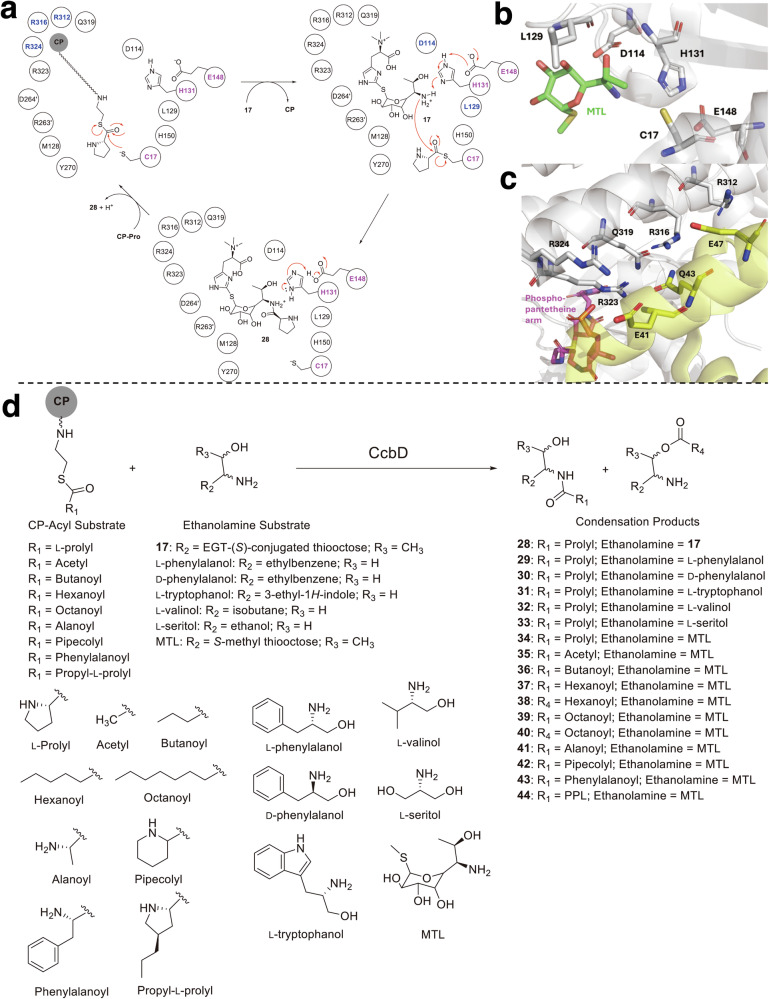
Structure-function analysis and substrate specificity of CcbD. **a** Proposed catalytic cycle of CcbD. Catalytic residues are highlighted in magenta and residues controlling substrate specificity in each step are highlighted in blue. **b** Active site of CcbD in the complex structure with MTL (PDB ID: 7YN2). **c** substrate promiscuity of CcbD. **d**, Active site of CcbD in the complex structure with CcbZ-CP (PDB ID: 7YN3). Residues from CcbD and CcbZ-CP are colored grey and yellow, respectively

In the subsequent step of the biosynthetic pathway, the condensation enzymes CcbD and LmbD catalyze amide bond formation between the thiooctose and alkylproline subunits in a carrier protein (CP)-dependent manner [37]. This reaction is reminiscent of the condensation domain of NRPSs, although the enzymes share little sequential and structural similarity to the domain. Instead, CcbD and LmbD employ a Cys-His-Glu catalytic triad resembling that of cysteine proteases in a ping-pong mechanism (Fig. 5a). In the CcbD reaction, the CP-tethered proline is first loaded onto the catalytic cysteine, and the resulting thioester is then attacked by the amino group of the thiooctose **17**, which is in turn activated by His and Glu, forming the amide bond of the condensation product **28**.

The complex structure of CcbD with MTL showed that the sugar moiety binding is mediated primarily by Asp114, Met128, Leu129, His131, and Tyr270, together with Arg263’ and Asp264’ from the adjacent monomer, through interactions with the hydroxy and amino groups of the thiooctose (Fig. 5b). The large binding pocket and the relaxed recognition of the ethanolamine moieties by Asp114, Leu129, and the catalytic His131 allow the accommodation of bulky amino-containing substrates, leading to the formation of dipeptide compounds (**29**-**33**). Unconjugated MTL is also accepted as a substrate to generate **34**, indicating that the *S*-substituted moiety is not essential for sugar recognition, as it faces toward the entrance of the active site (Fig. 5b and d).

In addition, the complex structure of CcbD with CcbZ-CP revealed an interaction network in which Arg312, Arg316, Gln319, Arg323, and Arg324 of CcbD engage Glu40, Glu41, Gln43, and Glu47 of CcbZ-CP. In celesticetin biosynthesis, CcbD normally incorporates proline in this CP–dependent manner, and CP recognition by Arg312, Arg316, and Arg324 is critical for proper substrate positioning and efficient amide bond formation (Fig. 5c). Nevertheless, CcbD is not limited to proline incorporation but displays broad acyl-substrate promiscuity, accepting a variety of fatty acyl and amino acyl groups to generate non-natural lincosamide analogues (**35**-**43**). Additionally, the enzyme can utilize an LmbN-CP-tethered propyl-L-proline to generate hybrid lincomycin-celesticetin products (**44**, Fig. 5d).

## Product specificity engineering of LmbF and CcbF

**Fig. 6:**
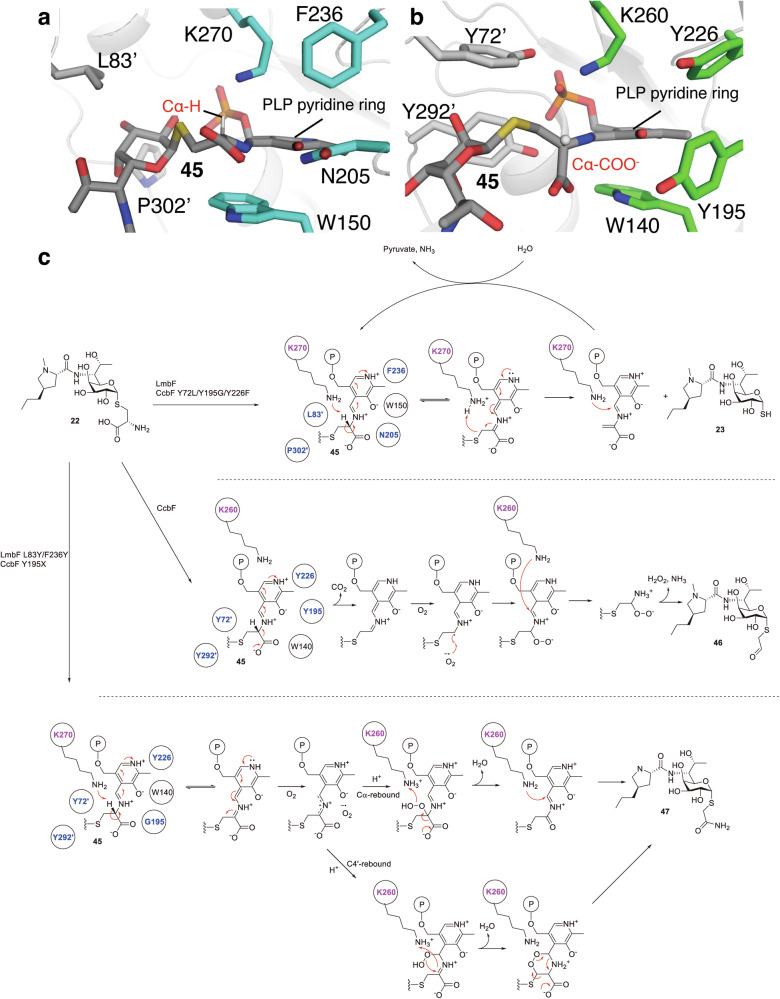
Catalyses of LmbF and CcbF. Binding mode of substrate in **a**, LmbF and **b**, CcbF. **c**, Proposed catalytic mechanisms of LmbF and CcbF. Catalytic residues are highlighted in magenta and residues governing reaction specificity are highlighted in blue

In the late stage of the biosynthetic pathways, the pyridoxal 5’-phosphate (PLP)-dependent enzymes LmbF and CcbF mediate the *S*-diversification of *S*-glycosyl-L-cysteine lincosamide intermediates such as **22**. Despite sharing ~40% amino acid sequence identity and recognizing the same cysteine-containing substrate, these enzymes catalyze distinct chemical transformations. LmbF mediates an oxygen-independent β-elimination to generate the thiol group in **23**, whereas CcbF catalyzes an oxidative deamination reaction accompanied by decarboxylation to form the *S*-acetaldehyde product **46** (Fig. 6).

To elucidate the structural basis underlying their divergent catalytic specificities, the crystal structures of LmbF and CcbF in complex with PLP were solved [38]. Comparison of their active sites revealed that while the PLP-binding mode is largely conserved, four residues positioned near PLP, Leu83, Asn205, Phe236, and Pro302 in LmbF, are replaced by tyrosine residues (Tyr72, Tyr195, Tyr226, and Tyr292, respectively) in CcbF. To investigate the importance of these residues, docking and molecular dynamics (MD) simulations were performed based on the crystal structures. The simulations indicated that the substrate binding modes differ substantially between the two enzymes. In LmbF, the Cα–H bond of the amino acid moiety of internal aldimine **45** is oriented nearly perpendicular ( ~ 90°) to the plane of the PLP pyridine ring, maintaining a parallel alignment with the π-system of the external aldimine intermediate, which is favorable for Cα–H bond cleavage and β-elimination to generate product **23** (Fig. 6a). In contrast, in CcbF, the bulky tyrosine residues protrude into the active site and reposition the substrate such that the Cα–COOH bond of **45** is aligned parallel to the π-system, which would facilitate decarboxylation and oxidative deamination, rather than β-elimination to generate product **46** (Fig. 6b and c).

Guided by these structural insights, site-directed mutagenesis was performed to alter the catalytic properties of CcbF. Notably, site-saturation mutagenesis at position 195 identified the CcbF Y195G variant, which generates a novel *S*-acetoamide lincosamide analogue **47** through an oxidative amidation reaction. Additional mutations of CcbF Y195G at Tyr72 and Tyr226 yielded the triple mutant CcbF Y72L/Y195G/Y226F, which exclusively produced the same β-elimination product as LmbF. This demonstrated the complete functional conversion of CcbF from its native oxidative deamination activity to the β-elimination activity of LmbF and the production of an unnatural novel lincosamide analogue. These results confirmed that a small set of active-site residues dictates the reaction specificity of these PLP-dependent enzymes. Future systematic structure–function analyses of diverse PLP-dependent enzymes are expected to clarify the general principles governing their catalytic selectivity, thereby enabling the rational design of engineered PLP-dependent biocatalysts for the generation of lincosamide analogues.

## Summary and outlook

More than half a century after their discovery, lincosamides continue to be valuable pharmaceuticals. The elucidation of lincomycin A biosynthesis has uniquely positioned this compound for the exploitation of its biosynthetic pathway. Recent studies have revealed that a complex network of transcriptional regulators controls the expression of biosynthetic genes through both direct and indirect mechanisms. Future studies on the interplay among these regulators will guide genetic engineering strategies to optimize antibiotic yields for industrial production. As the discovery of new antibiotic compounds becomes increasingly challenging, maximizing the potential of existing chemical classes is becoming more important. The lincosamide scaffold has recently regained attention as a valuable framework for drug development, because both natural and synthetic derivatives show strong biological activities. Moreover, lincosamide biosynthetic enzymes possess remarkable plasticity in their substrate and reaction specificities, making them attractive targets for protein engineering aimed at generating novel derivatives. Building on these insights, future efforts integrating rational design with experimental engineering are expected to pave the way for the development of next-generation antibiotics.
